# Metformin-treated cancer cells modulate macrophage polarization through AMPK-NF-κB signaling

**DOI:** 10.18632/oncotarget.14982

**Published:** 2017-02-01

**Authors:** Chi-Fu Chiang, Ting-Ting Chao, Yu-Fu Su, Chia-Chen Hsu, Chu-Yen Chien, Kuo-Chou Chiu, Shine-Gwo Shiah, Chien-Hsing Lee, Shyun-Yeu Liu, Yi-Shing Shieh

**Affiliations:** ^1^ Graduate Institute of Medical Sciences, National Defense Medical Center, Taipei, Taiwan; ^2^ Medical Research Center, Cardinal Tien Hospital, School of Medicine, New Taipei City, Taiwan; ^3^ Department of Radiation Oncology, Tri-Service General Hospital, National Defense Medical Center, Taipei, Taiwan; ^4^ Department of Dentistry, Tri-Service General Hospital, National Defense Medical Center, Taipei, Taiwan; ^5^ National Institute of Cancer Research, National Health Research Institutes, Miaoli, Taiwan; ^6^ Division of Endocrinology and Metabolism, Department of Internal Medicine, Tri-Service General Hospital, National Defense Medical Center, Taipei, Taiwan; ^7^ Department of Oral and Maxillofacial Surgery, Chi Mei Medical Center, Tainan, Taiwan; ^8^ Department of Biochemistry, National Defense Medical Center, Taipei, Taiwan

**Keywords:** breast cancer, macrophage polarization, metformin, microenvironment, NF-κB

## Abstract

Accumulating evidence is indicating metformin to possess the potential ability in preventing tumor development and suppressing cancer growth. However, the exact mechanism of its antitumorigenic effects is still not clear. We found that metformin suppressed the ability of cancer to skew macrophage toward M2 phenotype. Metformin treated cancer cells increased macrophage expression of M1-related cytokines IL-12 and TNF-α and attenuated M2-related cytokines IL-8, IL-10, and TGF-β expression. Furthermore, metformin treated cancer cells displayed inhibited secretion of IL-4, IL-10 and IL-13; cytokines important for inducing M2 macrophages. Conversely, M1 inducing cytokine IFN-γ was upper-regulated in cancer cells. Additionally, through increasing AMPK and p65 phosphorylation, metformin treatment activated AMPK-NF-κB signaling of cancer cells that participate in regulating M1 and M2 inducing cytokines expression. Moreover, Compound C, an AMPK inhibitor, significantly increased IL-4, IL-10, and IL-13 expression while BAY-117082, an NF-κB inhibitor, decreased expression. In metformin-treated tumor tissue, the percentage of M2-like macrophages decreased while M1-like macrophages increased. These findings suggest that metformin activates cancer AMPK-NF-κB signaling, a pathway involved in regulating M1/M2 expression and inducing genes for macrophage polarization to anti-tumor phenotype.

## INTRODUCTION

Diabetes and cancer are common diseases that significantly affect health across the globe. Several epidemiological studies have shown that people with diabetes are at significantly higher risk for cancer [1]. Type 2 diabetes and cancer share similar risk factors such as obesity, diet, alcohol consumption and smoking, and additionally, type 2 diabetes may influence carcinogenesis through mechanisms including hyperinsulinemia, hyperglycemia, and chronic inflammation [1]. Furthermore, several observational studies have suggested that some of the medications used to treat type 2 diabetes is associated with an increased or decreased risk of cancer.

The tumor microenvironment is now recognized as being an important factor for tumor progression and response to treatment [2]. Accumulating evidence suggest that the tumor microenvironment regulates tumor cells, thereby influencing malignancy and metastasis [3, 4]. A major characteristic of the tumor microenvironment is inflammatory cell infiltration. Macrophages are a major cellular component of tumors, where they are commonly termed tumor-associated macrophages (TAMs) [4]. TAMs are associated with poor prognosis and the development of a various tumor, including the lung, breast, prostate, glioma, bladder and lymph nodes [5]. The polarized state of TAMs can be classified into two macrophage subsets: classically activated (M1) and alternatively activated (M2) [6]. In nonmalignant or regressing tumors, most of the TAMs are of the classic subset (M1-like), representing pro-inflammatory activity, characterized by antigens and promotion of tumor lysis. In contrast, TAMs in malignant tumors tend to resemble the alternative subset (M2-like), which enhances tumor growth by producing cytokines and downregulating anti-tumor immune responses [4, 5]. A tumor is a complex microenvironment that differentially influences infiltrated macrophages, thus TAMs may be a target for new therapeutic molecules. The balance between M1 and M2 macrophages is a fundamental aspect of anti-tumor treatment, and the restoration of an M1 TAM phenotype may provide therapeutic value by promoting anti-tumor behavior.

Metformin is used to treat diabetes and is associated with a decreased risk of cancer and cancer-related mortality in diabetic patients [7]. It has a potential application to treat cancer by inhibiting cell proliferation thereby causing cell cycle arrest [8]. Many studies have been conducted to elucidate the underlying mechanism of the beneficial role of metformin in cancer. In cancer cells, AMPK activation by metformin has been shown to inhibit the mTOR pathway, global protein synthesis and proliferation in numerous different cancer cell lines [9]. Recent studies have shown that metformin may also target cancer-initiating cells [10] or repress the epithelial to mesenchymal transition [11]. However, the role of metformin in cancer therapy is controversial. Although the precise mechanism of the antitumor effect of metformin is still unknown, this effect may be the result of inhibiting other mechanisms in the tumor microenvironment such as those involving TAMs. Recent studies have shown that metformin inhibits lung cancer metastasis by blocking the M2-like polarization of macrophages [4].

In this study, we found that metformin indirectly affects TAM polarization by regulating the expressions of cancer-related cytokines. We used THP-1 macrophages cultured with breast cancer conditioned medium (CM) to investigate the mechanisms by which metformin affects the expression of cytokines in breast cancer cells and TAM polarization. The potential signal pathway in metformin-treated cancer cells was also examined. Finally, animal experiments were conducted to evaluate the effect of metformin on the progression of breast tumor and TAM distribution and polarization. We found that metformin is able to modulate the expressions of cancer-related cytokines, which contributed to changes in TAM polarization. These finding may be beneficial to the development of new anticancer treatment.

## RESULTS

### M2 induction ability was attenuated in metformin-treated cancer cells

**Figure 1:**
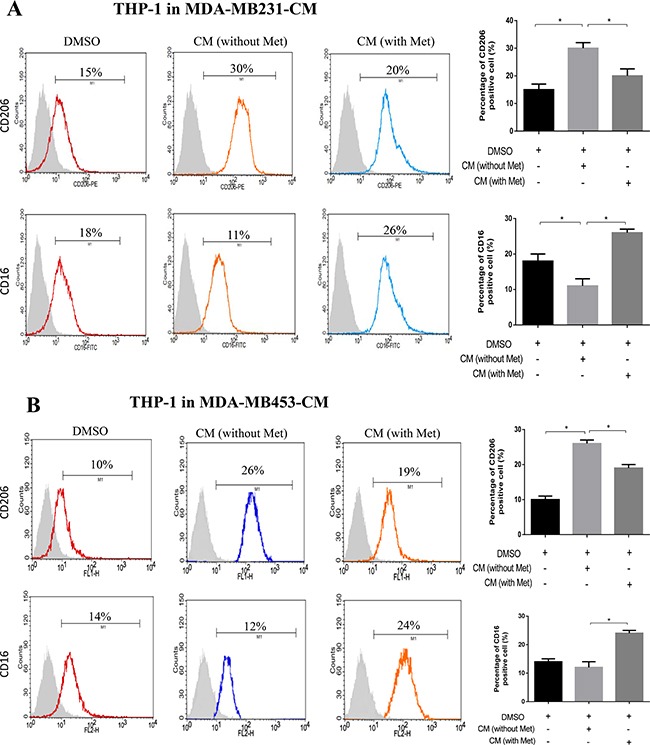
Metformin treated cancer cells polarized macrophage toward M1 phenotype THP-1 cells were stimulated with PMA (200 nM) for 24 h, then incubated with breast cancer (MDA-MB231/MDA-MB453) conditioned medium (CM) with or without metformin (60 μM) for 6 h, followed by flow cytometry analysis to quantify the amount of CD206, an M2 macrophage marker, and CD16, an M1 marker (**A, B**). Data are expressed as mean ± SD, *p < 0.05. DMSO: control; Met: metformin. Representative flow data shown are from experiments independently performed at least three times.

Breast cancer cells (MDA-MB231 and MDA-MB453) were treated with or without metformin (60 μM) for 6 h. The cultured medium was then replaced by fresh medium without serum, and 24 h later the CM was collected to treat THP-1 cells. Changes in the phenotype of the THP-1 cells was determined by examining their surface markers CD16 (M1) and CD206 (M2). Compared to the control group (DMSO), a significant increase in CD206 positive cells and decrease in CD16 positive cells was noted when the THP-1 cells were cultured in cancer CM (Figure 1A and 1B). This phenotype change was suppressed when the THP-1 cells were cultured in breast cancer CM pretreated with metformin (Met), in which a decrease in CD206 positive cells and increase in CD16 positive cells was noted compared to the group without metformin pretreatment (Figure 1A and 1B).

**Figure 2:**
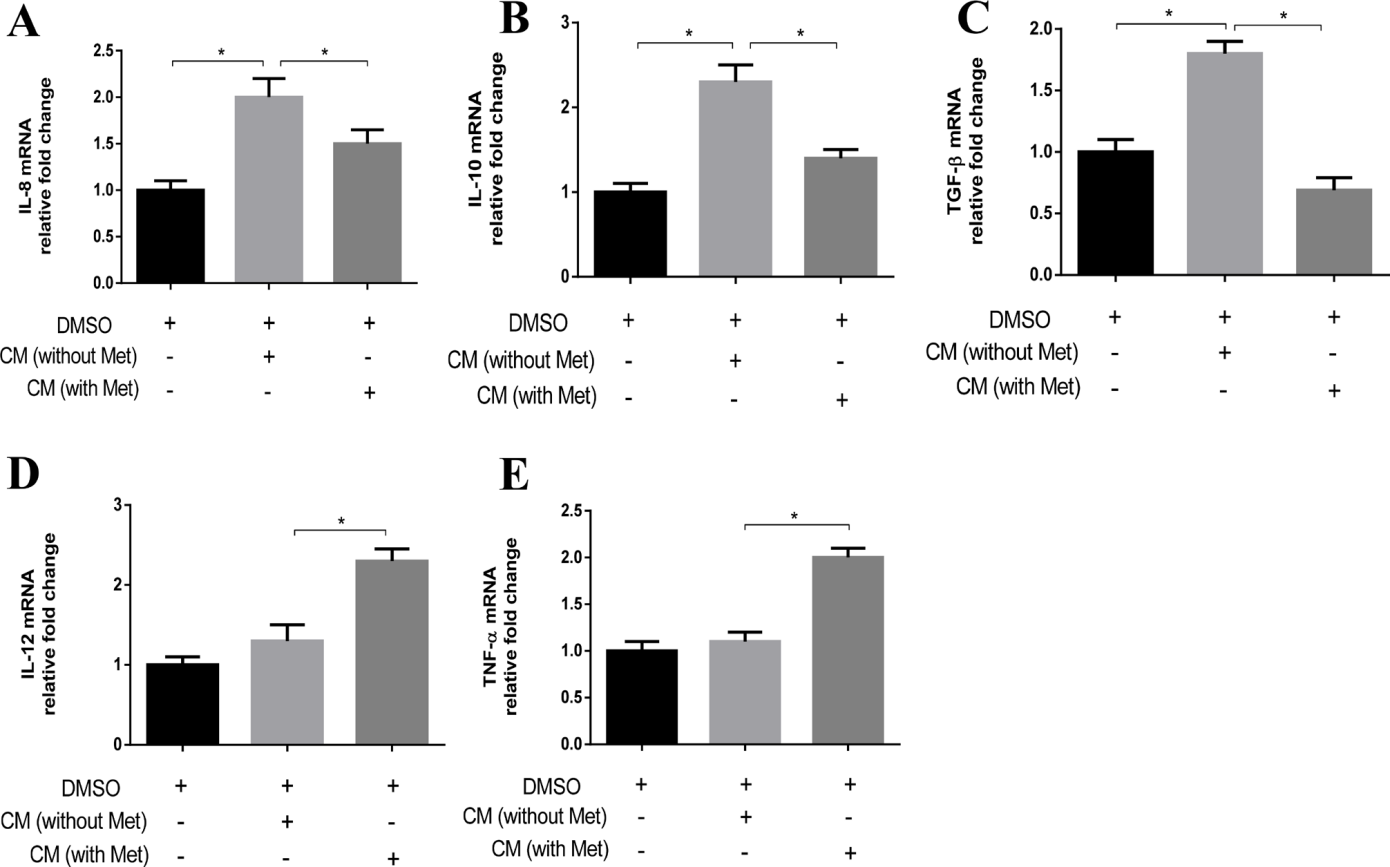
Metformin treated cancer cells increased M1 cytokine and decreased M2 cytokine expression in macrophage THP-1 cells were stimulated with PMA (200 nM) for 24 h, then incubated with breast cancer conditioned medium (CM) with or without metformin (60 μM) for 6 h, followed by analysis of the secretion of IL-8, IL-10, TGF-β, IL-12 and TNF-α using quantitative PCR (**A–E**). Data are expressed as mean ± SD, *p < 0.05. DMSO: control; Met: metformin. Representative quantitative PCR data shown are from experiments independently performed at least three times.

To further characterize these macrophages, we analyzed the expressions of M1- and M2-related cytokines in THP-1 cells cultured in the CM. After 24 h of treatment in the CM, the cells were washed and the medium was replaced with serum-free medium for another 24 h. The cells were then subjected to RNA extraction for gene expression analysis, and the medium was collected for ELISA analysis. M2-related genes such as IL-8, IL-10 and TGF-β were upregulated in the THP-1 cells cultured in the breast cancer cell CM compared to the controls (Figure 2A, 2B and 2C), whereas the expressions of M2-related genes were attenuated (Figure 2A, 2B and 2C) and the expressions of M1-related genes IL-12 and TNF-α were enhanced (Figure 2D and 2E) in the THP-1 cells cultured in the CM from breast cancer cells pretreated with metformin. Similar results were also observed for the expressions of these M1/M2 genes detected by ELISA (Supplementary Figure 1).

### Effects of metformin on cytokines secretion in breast cancer cells

**Figure 3:**
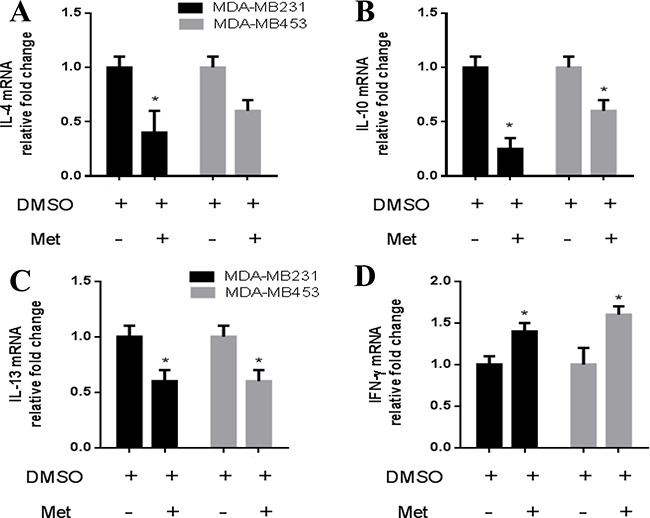
Metformin decreased IL-4, IL-10, IL-13 and increased IFN-γ expression in breast cancer cells Breast cancer cells (MDA-MB231/MDA-MB453) were treated with metformin (60 μM) for 6 h, followed by analysis of the secretion of IL-4, IL-10, IL-13 and IFN-γ using quantitative PCR (**A**–**D**). Data are expressed as mean ± SD, *p < 0.05. DMSO: control; Met: metformin. Representative quantitative PCR data shown are from experiments independently performed at least three times.

The M2 phenotype has been reported to be induced by cytokines including IL-4, IL-10 and IL-13, and the M1 phenotype has been reported to be induced by cytokines including IFN-γ [12]. Therefore, we analyzed whether the expressions of these cytokines were affected in cancer cells treated with metformin. We collected CM from breast cancer cells (MDA-MB231 and MDA-MB453) treated with or without metformin, and examined changes in the expressions of the genes of these cytokines. The expressions of IL-4, IL-10 and IL-13 were significantly decreased in the metformin-treated cancer cells compared to the control cells (Figure 3A, 3B and 3C). In contrast, the expression of IFN-γ was increased in the metformin-treated cancer cells (Figure 3D). Consistent with the RNA expression, protein secretion from the metformin-treated breast cancer cells also showed decreased expressions of IL-4, IL-10 and IL-13 in the medium (Supplementary Figure 2A, 2B and 2C) and an increased expression of IFN-γ (Supplementary Figure 2D).

### Metformin activated AMPK-NF-κB signaling in breast cancer cells

**Figure 4:**
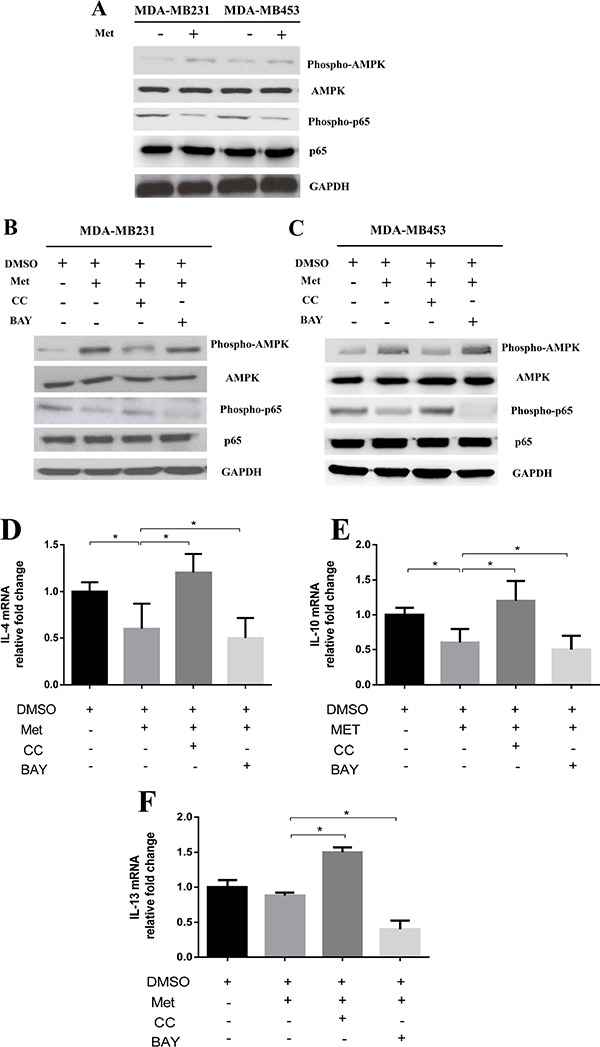
Metformin treatment activated AMPK and inhibited NF-κB signaling in cancer cells Breast cancer cells (MDA-MB231/MDA-MB453) were treated with metformin 60 μM for 6 h. The protein lysates were subjected to SDS-PAGE followed by immunoblotting with antibodies against phospho-AMPK, AMPK, phospho-p65, p65 and GAPDH (**A**). Breast cancer cells (MDA-MB231/MDA-MB453) were treated with metformin 60 μM combined with an AMPK inhibitor (Compound C, CC) or NF-κB inhibitor (BAY-117082, BAY) for 6 h. The protein lysates were subjected to SDS-PAGE followed by immunoblotting with antibodies against phospho-AMPK, AMPK, phospho-p65, p65 and GAPDH (**B, C**). The addition of the Compound C or BAY-117082 to metformin-treated cells and the secretion of IL-4, IL-10 and IL-13 from the breast cancer cells were assayed by quantitative PCR (**D, E, F**). Data are expressed as mean ± SD, *p < 0.05. DMSO: control; Met: metformin. Representative quantitative PCR data shown are from experiments independently performed at least three times.

A previous study reported that metformin-activated AMPK may participate in modulating the expressions of inflammatory cytokines through nuclear factor-κB (NF-κB) [13]. Therefore, we examined whether the expressions of cytokines in breast cancer cells treated with metformin were modulated through the AMPK-NF-κB pathway. We first examined the expressions of AMPK and NF-κB subunit p65 in breast cancer cells treated with metformin (60 μM) for 6 h. As shown in Figure 4A, metformin treatment increased the expression of phospho-AMPK and decreased the expression of phospho-p65 in the breast cancer cells. Next, we used an AMPK inhibitor (Compound C) and NF-κB inhibitor (BAY-117082) to further examine their role in the treatment effect of metformin. As shown in Figure 4B and 4C, Compound C attenuated metformin effect on p65 phosphorylation. Similarly, the expression of phospho-p65 was decreased by BAY-117082 in the metformin-treated breast cancer cells (Figure 4B and 4C). Metformin-induced AMPK phosphorylation did not appear to be altered by BAY-117082 (Figure 4B and 4C), suggesting that NF-κB is a downstream effector of phospho-AMPK.

We then evaluated whether AMPK-NF-κB signaling is involved in modulating the expressions of metformin-induced cytokines in breast cancer cells. As shown in Figure 4, the addition of Compound C to metformin-treated breast cancer cells significantly increased the expressions of IL-4, IL-10 and IL-13 when compared to metformin treatment alone (Figure 4D, 4E and 4F). Furthermore, the addition of BAY-117082 further decreased the expressions of these genes compared to metformin treatment alone (Figure 4D, 4E and 4F). Consistent with these results, the breast cancer cells treated with Compound C showed upregulated expressions of M2-induced cytokines IL-4, IL-10 and IL-13 (Supplementary Figure 3).

**Figure 5:**
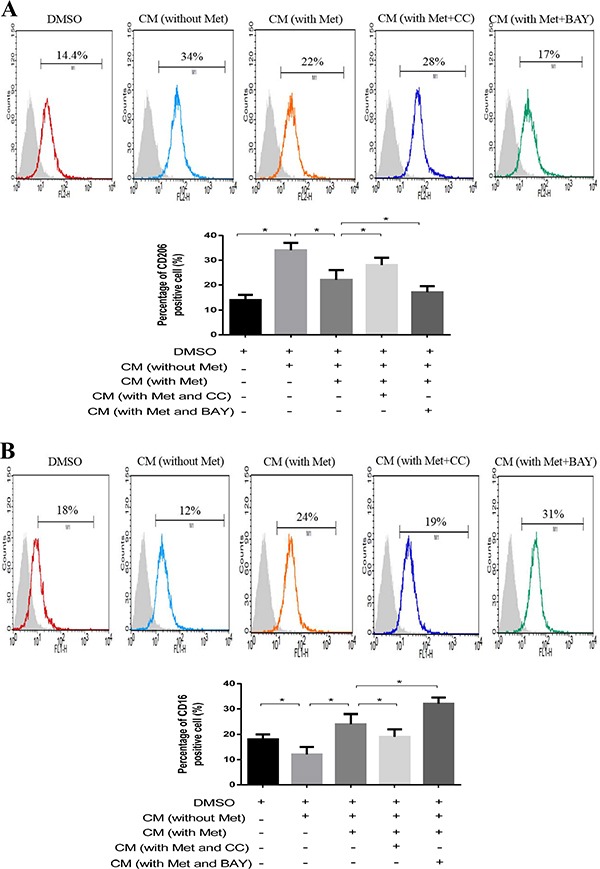
AMPK-NF-κB signaling participated in macrophage polarization Breast cancer cells (MDA-MB231) were treated with metformin 60 μM combined with an AMPK inhibitor (Compound C, CC) or NF-κB inhibitor (BAY-117082, BAY) for 6 h. The supernatant was collected to treat macrophages for 48 h, followed by flow cytometry analysis of CD206, M2 phenotype (**A**) and CD16, M1 phenotype (**B**). Data are expressed as mean ± SD, *p < 0.05. DMSO: control; CM: conditioned medium, Met: metformin. Representative flow data shown are from experiments independently performed at least three times.

Finally, we examined whether AMPK-NF-κB signaling in breast cancer cells was involved in the induction of macrophage polarization. CM from metformin-treated breast cancer cells with/without addition of Compound C or BAY-117082 was collected and used to culture THP-1 cells. As shown in Figure 5, the addition of Compound C increased the number of CD206 positive cells from 22% to 28% (Figure 5A) and decreased the number of CD16 positive cells from 24% to 19% compared to the cells treated with metformin alone (Figure 5B). In contrast, the addition of BAY-117082 significantly decreased the number of CD206 positive cells from 22% to 17% (Figure 5A) and increased the number of CD16 positive cells from 24% to 31% compared to the cells treated with metformin alone (Figure 5B).

### Effect of metformin treatment on breast tumor growth and TAM distribution *in vivo*

**Figure 6:**
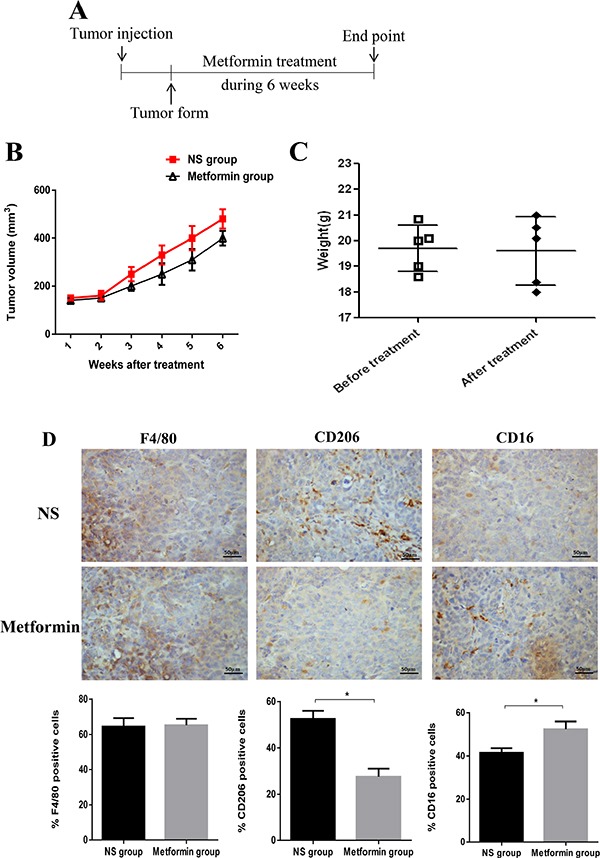
Administration of metformin affected tumor growth and TAM polarization in a xenograft model Schematic diagram of the experimental process in the xenograft model (**A**). The tumor volumes were determined (**B**). The weights of the mice were measured in all groups before and after metformin treatment (**C**). The tumor tissues were removed and subjected to immunohistochemistry (IHC) analysis. The infiltrated macrophages were analyzed for overall macrophage marker F4/80, M1 marker CD16, and M2 marker CD206, and the quantified data is shown. Scale bar 50 μm (**D**). Data are expressed as mean ± SD, *p < 0.05. NS: normal saline. Representative data are shown from experiments independently performed at least three times.

To further investigate the effect of metformin on tumor growth and TAM polarization *in vivo*, we injected breast cancer cells (MDA-MB231) subcutaneously into the flanks of nude mice. The tumor bearing mice were administered with metformin intraperitoneally (200 mg/ kg) three times a week, and then sacrificed (Figure 6A). The metformin-treated group demonstrated delayed tumor growth compared with the control group (Figure 6B and 6C). The tumors were then dissected and subjected to immunohistochemical staining for macrophage (F4/80), M1 (CD16), and M2 (CD206) specific antibodies. There was no significant difference in F4/80 positive macrophages between metformin-treated and control groups (Figure 6D). Of note, the number of CD16 positive macrophages was increased and the number of CD206 positive macrophages decreased in the metformin treatment group (Figure 6D). These results suggest that metformin treatment induced macrophage polarization toward the M1 phenotype *in vivo*. We then investigated whether metformin altered macrophage polarization through AMPK-NF-κB signaling in tumor tissue, and found that metformin treatment enhanced phospho-AMPK in the cytoplasm and decreased the expression of phospho-p65 in the nucleus (Supplementary Figure 4).

## DISCUSSION

In our study, we demonstrated that metformin exerts an anti-tumor effect through TAM polarization. First, we showed that metformin treatment can attenuate cancer cell polarization towards M2 phenotype through suppressing expression of M2 inducing cytokines. Secondly, we demonstrated that the effect of metformin on breast cancer cells could be regulated by cytokine expression via the AMPK-NF-κB pathway. Finally, *in vivo* experiments demonstrated metformin treated mice to have suppressed tumor growth and altered M1/M2 TAMs distribution. Taken together, our study provides both *in vitro* and *in vivo* evidence that the use of metformin in cancer therapy affects cancer cells directly and stroma cells such as TAMs indirectly. Therefore, regulating macrophage polarization may be an anti-cancer mechanism of metformin.

In this study, we found that metformin suppressed the progression of breast cancer by upregulating the expressions of M1 cytokines thereby polarized TAM toward M1. Previous studies have reported metformin to directly affect macrophage polarization, though results were controversial. For example, Ding et al. reported that metformin suppresses the IL-13-induced M2-like polarization of macrophages by reducing the expression of CD206 [4]. In the study of Chen et al., metformin induced single-cultured macrophages to an M2 phenotype, but attenuated M2 macrophage differentiation and inhibited the expression of M2-related cytokines when co-cultured with tumor cells [14]. In addition, Kim et al. reported that metformin inhibited lipopolysaccharides (LPS)-induced production of TNF-α and IL-6 in a concentration-dependent manner, but metformin alone, in the absence of LPS, had little effect on the macrophage production of TNF-α or IL-6 [15]. In our current study, we demonstrated that in addition to the direct effect on macrophage polarization, metformin also exerted an indirect effect to skew macrophages toward M1 polarization through modulating the expressions of cancer-related cytokines. Therefore, modulation of macrophage polarization by metformin is depended on the microenvironment.

TAMs exhibition of M2/M1 phenotype is dependent on the expression of a series of markers and cytokines. This study has chosen CD16 and CD206 as M1/M2 differential marker, respectively, as previous studies have consistently supported them as good indicators for these phenotypes [16–19]. CD16, a low-affinity IgG receptor, has been observed to be expressed on M1 macrophages with anti-tumoral cytokine IFN-γ expression, the characteristic function of M1 macrophage phenotype [20, 21]. Additionally, increased expression of CD16 cells was found to be significantly negatively correlated to tumor size and stage in breast cancer [22]. CD206, a 175-kDA type I transmembrane glycoprotein, has been reported to be absent in M1 macrophage expression and therefore is a good differential marker for M2 macrophages [23]. Previous studies in breast cancer model also showed the function of CD206 positive macrophages were more pro-tumoral M2 type [24]. Similarly, Zhang W al have reported macrophages lacking CD206 to exhibit up-regulation of pro-inflammatory cytokines such as IL-10 [17]. Actually, macrophage polarization is a dynamic process encompassing two extremes: the classically-activated M1 pathway and the alternative-activated M2 pathway [25]. Activation of these pathways and the subsequent variation of TAM subsets are dependent on the cytokine balance in the milieu [25]. Metformin-treated cancer CM was observed to lose the ability to induce M2 phenotype to macrophages while expression of M1 inducing cytokines was up-regulated. Previous studies have indicated TAM to be capable of expressing both M1 and M2 polarization markers [26, 27]. For example, Argianse-1, despite being considered a classic M2 marker, was found to be up-regulated in M1 macrophages [28, 29]. In metformin-treated cancer CM, the concurrent presence of M1 and M2 inducing cytokines could result in macrophages developing a mixed M1/M2 phenotype. As M1 and M2 signatures are not mutually exclusive and often coexist together, accurate assessment of macrophage polarization must include both cell surface markers and cell function, such as cytokine expression.

Our results also demonstrated that AMPK-NF-κB signaling in metformin-treated breast cancer cells participated in THP-1 polarization, where blockade of AMPK and NF-κB with specific inhibitors diminished the effect of metformin in breast cancer cells. AMPK is a serine/threonine protein kinase that acts as a central metabolic sensor involved in cellular energy homeostasis [30]. Recent studies have indicated that AMPK is not simply an energy sensor. The role of AMPK in cancer and whether it promotes or suppresses tumor growth remains controversial [31]. For example, Hadad et al. demonstrated reduced AMPK signaling in patients with breast cancer compared to strong expression in normal breast epithelium. In addition, reduced AMPK signaling was significantly associated with higher histological grade and axillary node metastasis [32]. Jang et al. found that AMPK was prominently expressed during neurocarcinogenesis, from the occurrence of early hyperplasia to the emergence of large gliomas [33]. Jhaveri et al. reported AMPK to regulate HER2 and EGFR activity in HER2-amplified breast cancer cells, and that HER2 and EGFR over-expressed cells were more sensitive to the cytotoxic effects of the AMPK activator [34]. AMPK is not a single peptide enzyme; rather it is a complex of three subunits chosen, in a given cell, from an available pool of seven subunits. Little is known about whether each complex localizes to a specific subcellular locale with a distinct situation-dependent function targeting a specific set of substrates, or whether the same AMPK complex functions differently in diseased tissue compared with normal tissue. Further studies on the conflicting results of AMPK function in healthy and diseased tissue may yield a deeper understanding of these complex issues.

Inflammation plays a key role in both cancer and diabetes, and altered glucose and energy metabolism is a major feature of cancer cells [30–32]. We therefore hypothesize that metformin targets the AMPK signal transduction pathway, ultimately reaching NF-κB and subsequently provoking an inflammatory response. By activating AMPK signaling, metformin inhibits inflammation and aspects of glucose and energy metabolism, and it is possible that additional links between inflammation and metabolism may reinforce the effects of metformin. While AMPK has been proposed to be a therapeutic target in breast cancer and inhibitor of many pathways regulated by tyrosine kinase growth factor receptors, our results provide evidence that metformin-activated AMPK directly phosphorylates and inhibits NF-κB activity. These important cell signaling interactions between AMPK and NF-κB have implications with respect to the prevention and treatment of cancer.

In addition to our cell line study, our *in vivo* study also showed that AMPK and NF-κB immunoactivity was correlated with the distribution and density of M2-type TAMs, as highlighted by the M2 macrophage marker CD206. Macrophages with combinations of M1 and M2 markers have been found in various disease processes. Further investigation is required regarding the contribution of coexisting macrophages with different phenotypes, the impact of dynamic changes of macrophage plasticity on diseases, and the molecular networks orchestrating the switch of macrophage phenotype in order to better understand the M1/M2 paradigm of macrophage polarization. According to the results of this study, we hypothesize that metformin-activated AMPK-NF-κB signaling in breast cancer participates in TAM polarization toward a M1 phenotype with an antitumor characterization.

## MATERIALS AND METHODS

### Cell culture and reagents

Breast cancer cell lines MDA-MB231 and MDA-MB453 were cultured in RPMI-1640 medium (Gibco, Grand Island, NY, USA) with 4.5 g/L glucose supplemented with 10% fetal bovine serum (FBS; Gibco BRL, Grand Island, NY, USA), 100 U/ml penicillin (Thermo, Wilmington, DE, USA), and 100 U/ml streptomycin (Thermo, Wilmington, DE, USA) under 5% CO_2_ at 37°C. The THP-1 cell line was obtained from Bioresource Collection and Research Center (BCRC, Taipei, Taiwan), and maintained in RPMI-1640 medium supplemented with 10% FBS under 5% CO_2_ at 37°C. The THP-1 cells were differentiated by 200 nM PMA for 24 h (phorbol 12-myristate 13-acetate) (Sigma-Aldrich, St Louis, MO, USA). Metformin, Compound C (AMPK inhibitor), and BAY-117082 (NF-κB inhibitor) (Sigma-Aldrich, St Louis, MO, USA) were obtained for cell proliferation and *in vitro* and *vivo* studies.

### Preparation of conditioned media from breast cancer cells

Cancer cells were seeded at a density of 1 × 10^4^ cells/cm^2^ for 72 h, and when cultures reached 80–90% confluence, cells were treated with or without metformin (60 μM) for 6 h. The medium was then replaced with fresh serum-free medium for 24 h, whence centrifugation was performed at 2,000 g at 4°C for 10 min to remove cell debris, followed by filtration with a 0.22-mm filter (Millipore, Billerica, MA, USA). The conditioned media was preserved at –80°C for further study.

### Quantitative PCR analysis

Total RNA was isolated using Trizol (Invitrogen, Grand Island, NY, USA). Complementary DNA synthesis was performed using a SuperScript^®^ III Reverse Transcriptase kit (Invitrogen, Grand Island, NY, USA). Real-time PCR for the genes of interest was dyed with SYBR green (Thermo, Wilmington, DE, USA) using a LightCycler^®^ 480 System (Roche, California, USA). The primer sequences are listed in Table 1. The reaction mixture containing reverse transcribed cDNAs was preheated for 7 min at 95°C to activate the Taq polymerase. Forty cycles of PCR, each consisting of a 10-s denaturation step at 95°C, a 30-s annealing step at 60°C (two-step RT-PCR) were then performed. Throughout the real-time PCR analysis, the identity of the products was confirmed by melting curve analysis. The ratio of the amount of target mRNA to the amount of the internal standard (GAPDH) mRNA was determined as an arbitrary unit.

**Table1:** Primers used for qRT-PCR analysis

Genes		primer sequence(5′→3′)
IL-4	Forward primer	CCGTAACAGACATCTTTGCTGCC
	Reverse primer	GAGTGTCCTTCTCATGGTGGCT
IL-8	Forward primer	GAGAGTGATTGAGAGTGGACCAC
	Reverse primer	CACAACCCTCTGCACCCAGTTT
IL-10	Forward primer	TCTCCGAGATGCCTTCAGCAGA
	Reverse primer	TCAGACAAGGCTTGGCAACCCA
IL-12	Forward primer	TGCCTTCACCACTCCCAAAACC
	Reverse primer	CAATCTCTTCAGAAGTGCAAGGG
IL-13	Forward primer	ACGGTCATTGCTCTCACTTGCC
	Reverse primer	CTGTCAGGTTGATGCTCCATACC
TNF-α	Forward primer	CTCTTCTGCCTGCTGCACTTTG
	Reverse primer	ATGGGCTACAGGCTTGTCACTC
TGF-β	Forward primer	TACCTGAACCCGTGTTGCTCTC
	Reverse primer	GTTGCTGAGGTATCGCCAGGAA
IFN-γ	Forward primer	AGCTCTGCATCGTTTTGGGTT
	Reverse primer	GTTCCATTATCCGCTACATCTGAA
GAPDH	Forward primer	CCACATCGCTCAGACACCAT
	Reverse primer	TGACCAGGCGCCCAATA

### Western blotting

Whole cell lysates for Western blotting were harvested in RIPA buffer (1% SDS and 10 mM Tris buffer pH 7.4) containing protease inhibitors and phosphatase inhibitor (Thermo, Wilmington, DE, USA). Protein concentrations in the supernatants were determined using a Pierce BCA Protein Assay kit (Thermo, Rockford, IL, USA). Thirty micrograms of protein were separated on 5–15% gradient SDS-PAGE gel and transferred to polyvinylidene difluoride membranes (Millipore, Bedford, MA, USA) by wet blotting using an electroblotter (Hoefer, Holliston, MA, USA). Membranes were blocked for 1 h at 25°C with 2% bovine serum albumin or 5% skimmed milk in Tris-buffered saline Tween 20 (TBST). The membranes were incubated with appropriate dilutions of the primary antibodies: AMPK antibody (3694-S [1:1000 dilution]; Abcam, Cambridge, UK), phospho-AMPK antibody (2802-S (Thr172) [1:1000 dilution]; Abcam, Cambridge, UK), phospho-p65 antibody (3033 (Ser536) [1:1500 dilution]; Cell Signaling, Danvers, MA, USA), p65 antibody (3034 [1:1500 dilution]; Cell Signaling, Danvers, MA, USA), GAPDH antibody (ab8245 [1:2000 dilution]; Abcam, Cambridge, UK), overnight at 4°C. After being washed in TBST three times, the membranes were incubated for 60 minutes with HRP-conjugated goat anti-rabbit or anti-mouse secondary antibodies at 25°C. Specific bands were detected by chemiluminescence, and visualization was performed by exposing the membranes to RX film. The Western blotting experiments were repeated at least three times.

### Enzyme-linked immunosorbent assay (ELISA)

The THP-1 cells (5 × 10^4^ cells per well in a 96-well plate) were incubated with breast cancer conditioned medium with or without metformin, and breast cancer cells (5 × 10^4^ cells per well in a 96-well plate) were treated with or without metformin. After incubation for 48 h, the supernatants were collected, and levels of human IL-4, IL-8, IL-10, IL-12, IL-13, TNF-α, TGF-β, and IFN-γ were determined using an ELISA Ready-Set-Go kit (eBioscience, San Diego, USA) according to the manufacturer's instructions. Each experiment was performed in triplicate and repeated twice to assess the consistency of the results.

### Flow cytometry

THP-1 cells (1 × 10^6^ cells per well of 6-well plate) were incubated with breast cancer conditioned medium with or without metformin. After incubation for 48 h, the cells were collected with a scraper and blocked with incubation buffer (0.5 g bovine serum albumin in 100 ml PBS) for 45 min, followed by incubation with FITC-conjugated CD16 antibody (11–0168 [1:1000 dilution] eBioscience, San Diego, USA), PE-conjugated CD206 antibody (12–2069 [1:1000 dilution] eBioscience, San Diego, USA) for 60 minutes at 25°C. Following the final washing step, labeled cells were analyzed by flow cytometry on a FACScan flow cytometer using CellQuest software (Becton-Dickinson, Franklin Lakes, NJ). A total of 2 × 10^5^ cells was harvested at a collection speed of 200–300 cells/sec. Each experiment was performed in triplicate and repeated twice to assess the consistency of the results.

### Animal experiment

C57BL/6-background athymic nude mice (4–5 weeks old) were purchased from the National Laboratory Animal Center, Taiwan. The mice were raised according to institutional guidelines approved by the National Defense Medical Center of the Laboratory Animal Center (NLAC, Taiwan, ROC). MDA-MB231 cells were injected subcutaneously (1 × 10^6^ cells in 0.1 mL serum-free medium per mouse) into the flanks of the mice. The mice were randomly divided into two groups including an experimental group and control group when the tumors had reached an average volume of 100–130 mm^3^. The tumor-bearing nude mice were given metformin (200 mg/kg) intraperitoneally three times a week, and the control group was injected with normal saline. The body weight and tumor volume of the mice were monitored once a week. After the sixth measurement on day 42, the mice were sacrificed and the tumors removed and weighed. The tumors were then fixed in 4% paraformaldehyde and embedded in paraffin for the histological examination. The tumor volumes were measured using the following formula: (A × B^2^)/2, where A is the length and B is the width of the tumor. All experiments were repeated twice.

### Immunohistochemistry

Tumor sections were deparaffinized and rehydrated before antigen retrieval was performed by heating the tissue sections in 0.01 mol/L (pH 6.0) citrate buffer for 20 min in a microwave oven. Endogenous peroxidase and alkaline phosphatase activity were blocked with 3% H_2_O_2_ for 10 min. The tissue sections were then incubated at 4°C overnight with primary antibodies: F4/80 antibody (MCA497GA [1:400 dilution] AbD Serotec, Hercules, USA ), CD206 antibody (MCA2235EL [1:500 dilution] AbD Serotec, Hercules, USA), CD16 antibody (MCA5998 [1:500 dilution] AbD Serotec, Hercules, USA), phospho-AMPK antibody (2802-S (Thr172) [1:500 dilution]; Abcam, Cambridge, UK), phospho-p65 antibody (3033 (Ser536) [1:500 dilution]; Cell Signaling, Danvers, MA, USA), following the manufacturers’ recommendations. After washing three times, a secondary biotinylated antibody was added for 30 min at 25°C, followed by diaminobenzidine as a chromogen. These tissue sections were lightly counterstained with hematoxylin and examined under an optical microscope. Cells were counted in five randomly selected fields for each section. The immunohistochemistry results were analyzed using Image-Pro Plus software (Media Cybernetics, Crofton, MA, USA).

### Statistical analysis

Data were expressed as the mean ± SD (standard deviation) unless otherwise noted. Differences between groups were analyzed using a two-tailed Student's *t*-test when only two groups were compared, and the null hypothesis was rejected at the 0.05 level.

## SUPPLEMENTARY MATERIALS FIGURES



## Abbreviations

AMPK: AMP-activated protein kinase
CC: Compound C
CM: Conditioned Medium
IFNγ: Interferon gamma
LPS: Lipopolysaccharides
mTOR: mechanistic target of rapamycin
NF-kB: NF-kappaB
PMA: phorbol 12-myristate 13-acetate
TNF-α: Tumor necrosis factor-alpha
TAMs: Tumor-associated macrophages
TGF-β: Transforming growth factor-beta
VEGF: Vascular endothelial growth factor
