# Supplementary material for: Metformin-treated cancer cells modulate macrophage polarization through AMPK-NF-κB signaling
Source: Oncotarget. 2017 Feb 1;8(13):20706–18. doi: 10.18632/oncotarget.14982 (PMC5400538; doi:10.18632/oncotarget.14982)
Supplement: Supplementary file 1 [file oncotarget-08-20706-s001.pdf]

Metformin-treated cancer cells modulate macrophage polarization through AMPK-NF-κB signaling

Supplementary Materials

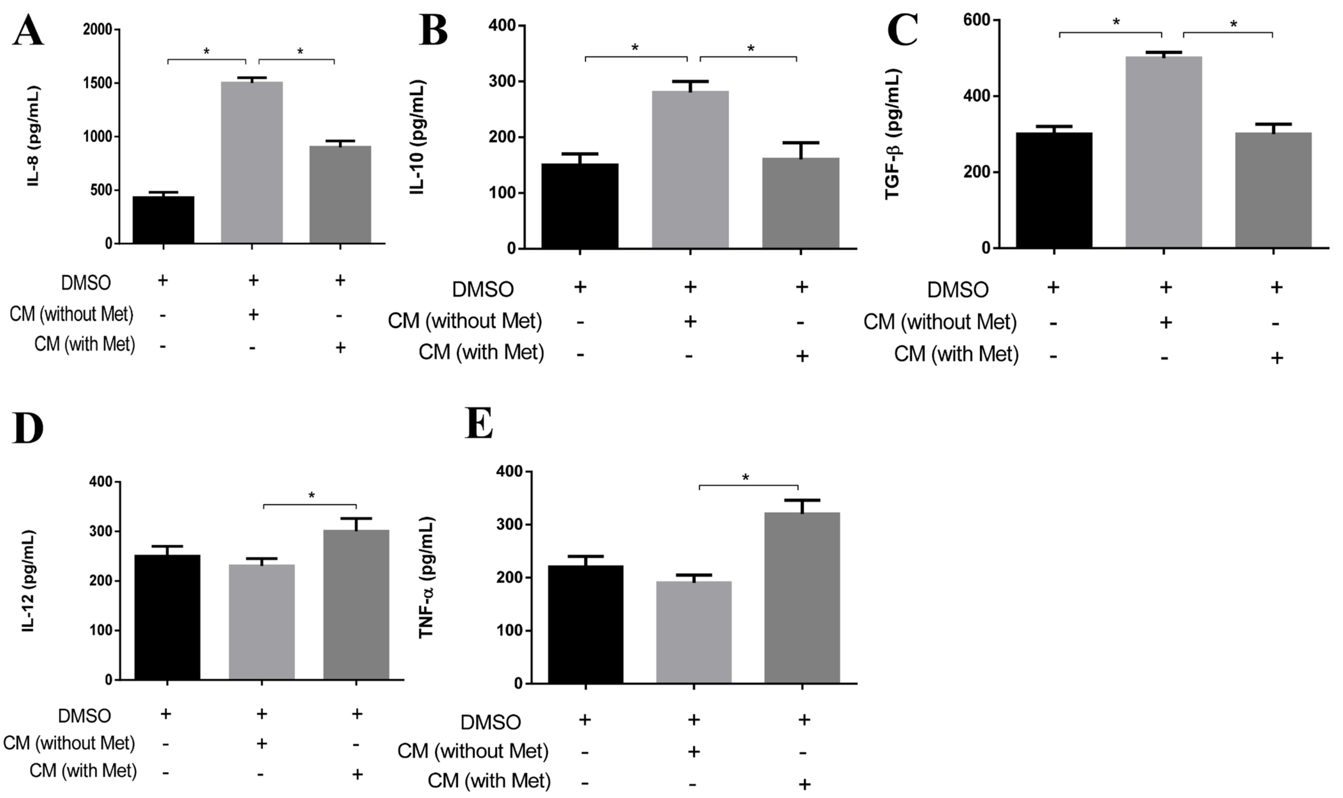

Supplementary Figure 1: Metformin treated cancer increased M1 cytokine and decreased M2 cytokine expression in macrophage. THP-1 cells were stimulated with PMA (200 nM) for 24 h, then incubated with breast cancer conditioned medium (CM) with or without metformin (60 μM) for 6 h, followed by ELISA analysis of IL-8, IL-10, TGF-β, IL-12 and TNF-α secretions (**A–E**). Data are expressed as mean ± SD, *$p < 0.05$. DMSO: control; Met: metformin. Representative ELISA data shown are from experiments independently performed at least three times.

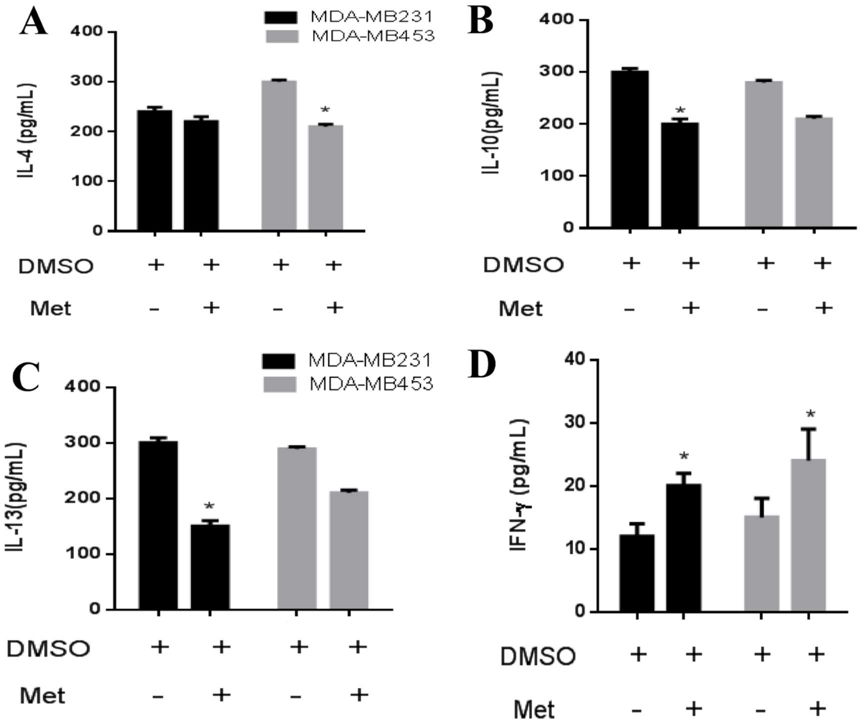

Supplementary Figure 2: Metformin decreased IL-4, IL-10, IL-13 and increased IFN-γ expression in breast cancer cells. Breast cancer cells (MDA-MB231/MDA-MB453) were treated with metformin (60 μM) for 6 h, followed by analysis of the secretion of IL-4, IL-10, IL-13 and IFN-γ using ELISA (**A–D**). Data are expressed as mean ± SD, *p < 0.05. DMSO: control; Met: metformin. Representative ELISA data shown are from experiments independently performed at least three times.

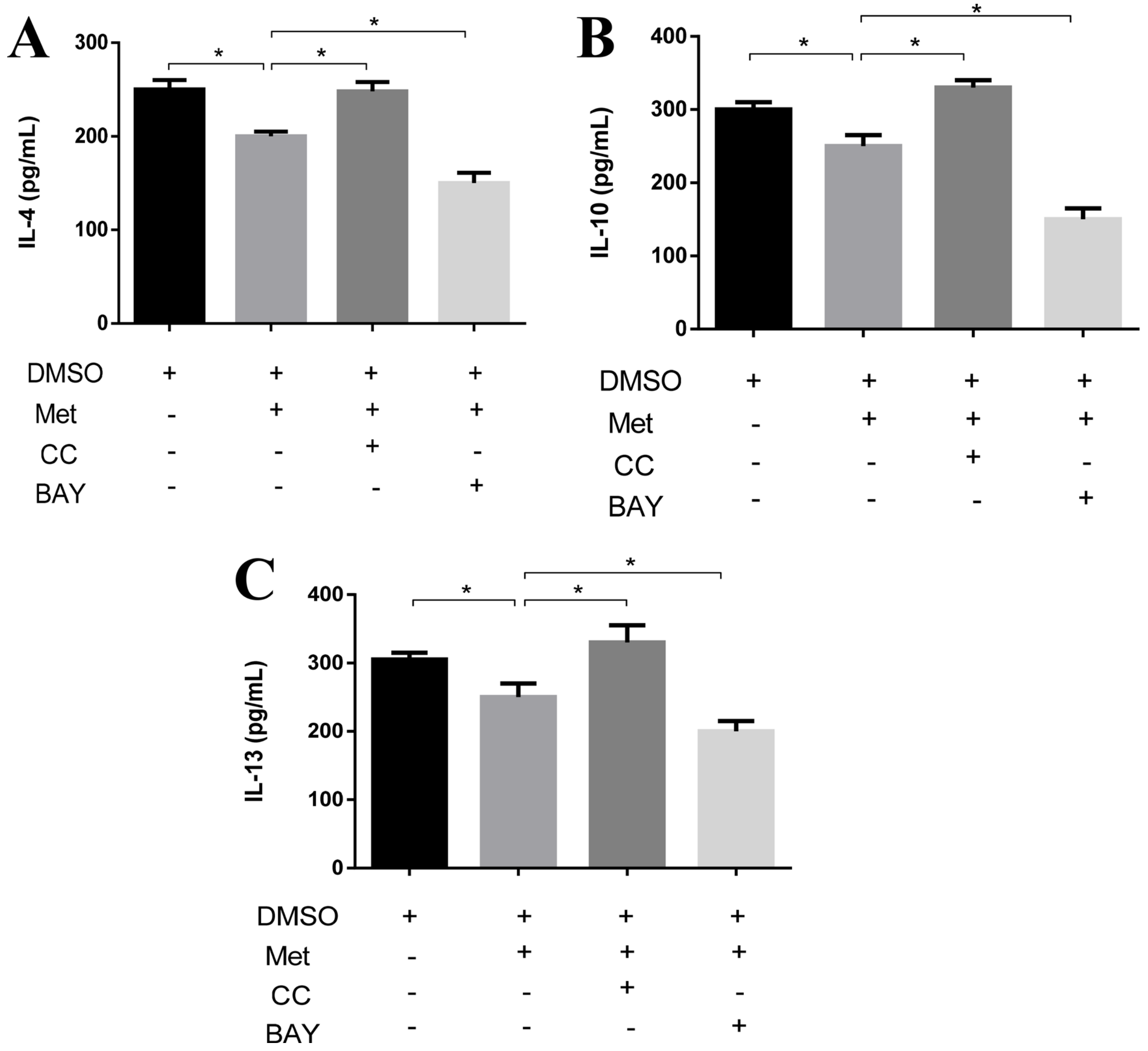

Supplementary Figure 3: Metformin treatment activated AMPK and inhibited NF-κB signaling in cancer cells. Breast cancer cells (MDA-MB231) were treated with metformin 60 μM for 6 h. After metformin incubation with an AMPK inhibitor (Compound C, CC) or NF-κB inhibitor (BAY-117082, BAY), the secretion of IL-4, IL-10 and IL-13 from breast cancer cells was measured by ELISA (**A**–**C**). Data are expressed as mean ± SD, $*p < 0.05$. DMSO: control; Met: metformin. Representative ELISA data shown are from experiments independently performed at least three times.

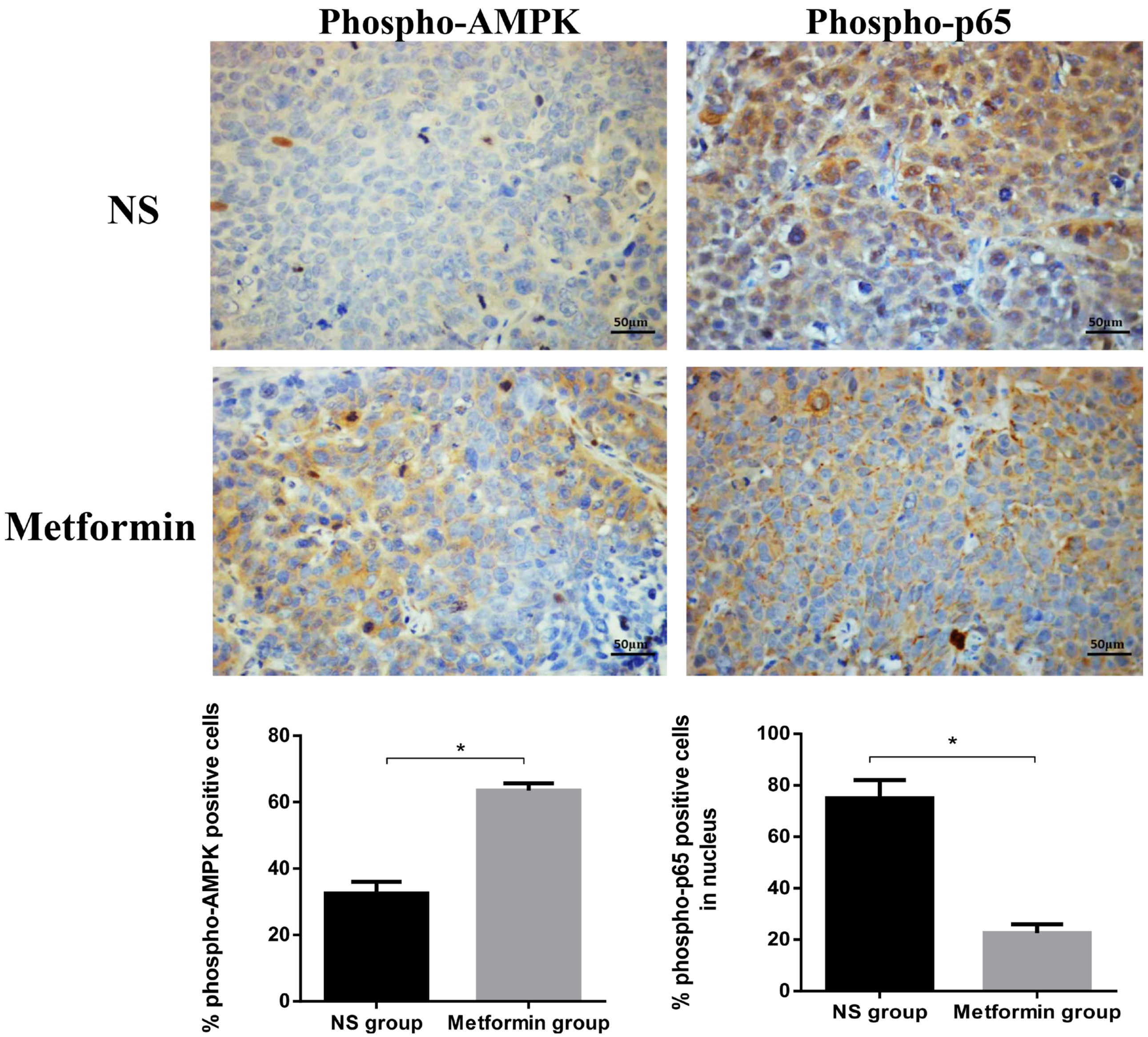

Supplementary Figure 4: Administration of metformin affected tumor growth and TAM polarization in a xenograft model. Schematic diagram of the xenograft model experimental process (A). The tumor tissues were removed and subjected to immunohistochemistry (IHC) analysis. The infiltrated macrophages were analyzed for phospho-AMPK and phospho-p65. Scale bar 50 μm. Data are expressed as mean ± SD, $*p < 0.05$. NS: normal saline. Representative data shown are from experiments independently performed at least three times.
